# Influence of Kartogenin on Chondrogenic Differentiation of Human Bone Marrow-Derived MSCs in 2D Culture and in Co-Cultivation with OA Osteochondral Explant

**DOI:** 10.3390/molecules23010181

**Published:** 2018-01-16

**Authors:** Timea Spakova, Jana Plsikova, Denisa Harvanova, Marek Lacko, Stefan Stolfa, Jan Rosocha

**Affiliations:** 1 Associated Tissue Bank of Faculty of Medicine of P. J. Safarik University and L. Pasteur University Hospital, Trieda SNP 1, 04011 Kosice, Slovakia; jana.plsikova@upjs.sk (J.P.); denisa.harvanova@upjs.sk (D.H.); jan.rosocha@upjs.sk (J.R.); 2 Department of Orthopaedics and Traumatology of Faculty of Medicine of P. J. Safarik University and L. Pasteur University Hospital, Trieda SNP 1, 04011 Kosice, Slovakia; marek.lacko@upjs.sk (M.L.); stefan.stolfa@unlp.sk (S.S.)

**Keywords:** kartogenin, mesenchymal stromal cells, osteochondral cylinder, osteoarthritis, chondrogenic differentiation

## Abstract

Articular cartilage has limited capacity for natural regeneration and repair. In the present study, we evaluated kartogenin (KGN), a bioactive small heterocyclic molecule, for its effect on in vitro proliferation and chondrogenic differentiation of human bone marrow-derived mesenchymal stromal cells (hBMSCs) in monolayer culture and in co-culture models in vitro. OA osteochondral cylinders and hBMSCs were collected during total knee replacement. The effect of KGN on hBMSCs during 21 days of culture was monitored by real-time proliferation assay, immunofluorescence staining, histological assay, scanning electron microscopy (SEM) (imaging and multiplex enzyme-linked immunosorbent assay) ELISA assay. The rate of proliferation of hBMSCs was significantly increased by treatment with 10 µM KGN during nine days of culture. Histological and SEM analyses showed the ability of hBMSCs in the presence of KGN to colonize the surface of OA cartilage and to produce glycosaminoglycans and proteoglycans after 21 days of co-culture. KGN treated hBMSCs secreted higher concentrations of TIMPs and the secretion of pro-inflammatory molecules (MMP 13, TNF-α) were significantly suppressed in comparison with control without hBMSCs. Our preliminary results support the concept that 10 µM KGN enhances proliferation and chondrogenic differentiation of hBMSCs and suggest that KGN is a potential promoter for cell-based therapeutic application for cartilage regeneration.

## 1. Introduction

Osteoarthritis (OA) is a progressively destructive joint disorder that causes articular cartilage erosion and fibrillation, as well as subchondral bone changes, including sclerosis, bone cysts, osteophytes, and synovitis. This socially-prevalent disease is characterized by complex interactions of cartilage, bone, the synovium, and systemic factors [1]. Articular cartilage represents an avascularised and non-innervated tissue with low cell mobility and a decreased number of progenitor cells, and has a limited capacity to self-repair. Therefore, cartilage defects and OA remain a major clinical challenge. Considerable efforts have been made to develop suitable cartilage repair procedures resulting in defect filling and restoration of the articular surface with the best possible tissue repair. There is evidence to support the idea that application of ex vivo expanded chondrocytes or mesenchymal stromal cells (MSCs) are relevant for cell-based therapies [2,3]. However, use of chondrocytes has several drawbacks such as a two-stage surgical procedure that may cause further cartilage damage and degeneration and chondrocyte dedifferentiation during in vitro culture. Among the cell sources that have the ability to regenerate cartilage, MSCs are considered a most promising cell type for the repair of damaged cartilage, especially due to their multilineage differentiation, immunosuppressive and immunomodulatory capacity via cell to cell interaction, or secretion of various factors.

One of the main problems in cartilage regenerative medicine is the formation of fibrocartilage with inferior properties than native hyaline cartilage. In order to further optimize cell-based therapies there is a need to stimulate MSCs towards extracellular matrix producing chondrocytes and keep them in their chondrogenic differentiation state [4]. The induction and regulation of MSCs’ chondrogenic differentiation in vivo involve a combination of multiple factors, like stem cell intrinsic factors, paracrine factors from neighbouring cells and microenvironmental components. In vivo studies of MSC implantation demonstrate that the microenvironment is key in inducing cartilage formation and maintenance of the cartilage phenotype [5]. Independent from cells, chondrogenic differentiation of MSCs and the deposition of a typical hyaline articular cartilage extracellular matrix (ECM) can be induced by stimulation with growth factors, like transforming growth factor-beta (TGF-β) superfamily members, bone morphogenic proteins (BMPs), or insulin-like growth factor (IGF) used in recently-published in vitro and in vivo studies [6,7]. However, growth factors not only induce a significant chondrogenic phenotype, but could also lead to further hypertrophic differentiation and contribute to the development of unwanted fibrous cartilage [8,9]. While these factors used in defined chondrogenic medium are well-understood and studied over a long-time, the factors responsible for the formation and maintenance of stable articular cartilage are still poorly defined.

Recently, a small heterocyclic molecule, kartogenin (KGN) (MW 317.34), has been discovered to enhance chondrogenic differentiation of human MSCs, by upregulating the expression of collagen II, Sox 9, and aggrecan with no significant effect on the gene products associated with chondrocyte calcification and hypertrophy (osteocalcin, collagen X) [10]. Interestingly, this molecule also has chondroprotective properties in cartilage explant cultures grown in the presence of tumour necrosis factor-α (TNF-α) and oncostatin M by blocking the degradation of both CD44 and aggrecan [11].

While the effect of KGN on cartilage formation has been reported previously in several in vivo studies using animal models [12,13,14,15,16], its effect on OA cartilage was not tested in clinical trials. Before being translated into clinical practice, it is necessary to evaluate the effect of KGN on cell proliferation and differentiation of hBMSCs in vitro. Recent studies have demonstrated that the ability of MSCs to differentiate into a specific cell phenotype is critically dependent on the surrounding environment [17], therefore, use of osteochondral explants in vitro instead of isolated chondrocytes resembles the natural structure and properties of native cartilage and promote cell-to-cell contact.

The aim of the present study was to establish an in vitro co-culture model system, where at least one cell type (chondrocyte) is kept in its original 3D matrix environment and evaluate the effect of KGN on cell proliferation and chondrogenic differentiation of hBMSCs in a co-culture model and in a monolayer culture, respectively. We assume that KGN, due to its unique properties, could become an effective accelerant for cartilage regeneration by promoting chondrogenic differentiation of human mesenchymal stromal cells.

## 2. Results

### 2.1. Characterization and Proliferation of hBMSCs

The human BMSC cells were characterized for their ability to adhere, express typical mesenchymal stromal cell markers, and differentiate into three mesodermal lineages. Throughout the culture period (until passage 2), the hBMSCs cultured in standard culture medium grew as monolayers and exhibited a characteristic spindle-like, fibroblastic morphology (Figure 2A). The donor hBMSCs used in this study showed that >95% of the cell population expressed CD73 (99.81%), CD90 (99.38%), and CD105 (98.77%), and <2% expressed the mixture of hematopoietic markers CD45/CD34/CD20/CD14 (1.95%). The functional characterizations of hBMSCs included chondrogenic, osteogenic, and adipogenic in vitro differentiation assays (data not shown). After three-week chondrogenic differentiation, the cells were stained for PGs by Alcian blue. The osteogenically-differentiated cells in the monolayer culture were stained by Alizarin red, and fatty droplets produced during adipogenic differentiation were visualized by Oil Red O staining.

**Figure 1:**
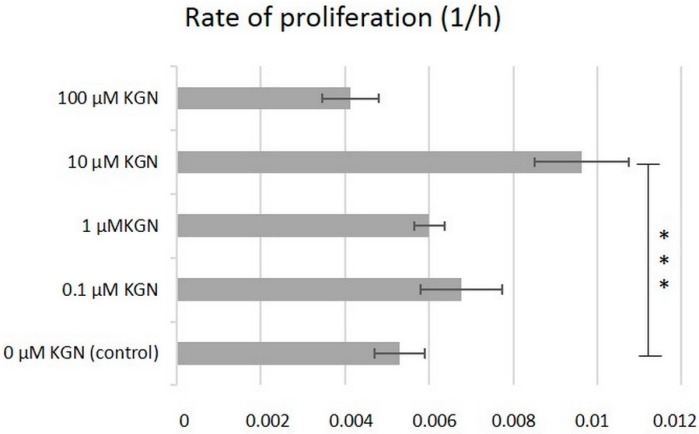
Effect of KGN on therate of proliferation of hBMSCs in monolayer culture during nine days. Values are expressed as mean ± SD (*n* = 4). *** *p* < 0.001 vs. the control group (hBMSCs in culture medium).

Primarily, the effect of increasing concentration of KGN on proliferation of hBMSCs from passage 2 was evaluated in this study using an automated xCelligence^®^ system (Roche Applied Science, Penzberg, Germany) (Figure 1).

The rate of proliferation of hBMSCs in culture medium supplemented with 0.1, 1, and 100 µM KGN was not affected significantly in comparison with the control without KGN (*p* < 0.05). hBMSCs cultured with 10 µM KGN showed the highest cell index during nine days of culture and the rate of proliferation significantly increased compared to the control (*p* < 0.001).

### 2.2. Cytoskeleton of hBMSCs in Monolayer during Chondrogenic Differentiation

**Figure 2:**
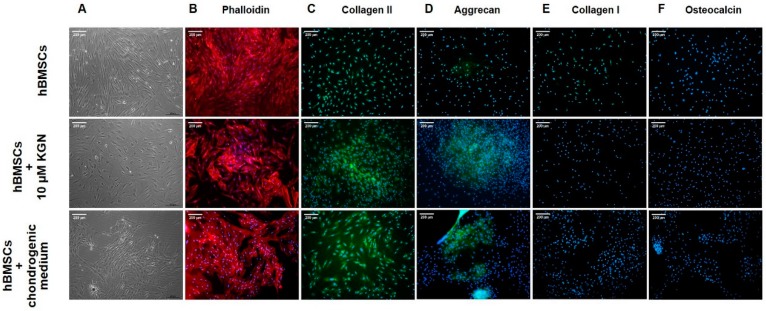
The effect of KGN on hMBSCs morphology and expression profile. Cell morphology and cytoskeleton of hBMSCs in monolayer culture during chondrogenic differentiation at day 21. Representative images of (**A**) phase contrast microscopy and (**B**) fluorescently-stained actin cytoskeleton of primary cultures of hBMSCs at passage 2. Effect of KGN on cartilage-specific and nonspecific markers detected by immunofluorescence in monolayer cultures of hBMSCs at day 21. (**C**) Cells were stained for collagen II (green); (**D**) cells were stained for aggrecan (green); (**E**) cells were stained for collagen I (green); and (**F**) cells were stained for osteocalcin (green). Cell nuclei were counterstained with DAPI (blue). Scale bar represents 200 µm.

Changes in cell shape during chondrogenic induction in monolayer culture were examined by using direct immunofluorescence. In basal culture medium hBMSCs exhibited a characteristic fibroblast-like morphology with long, parallel, thin stress fibres across the entire cytoplasm (Figure 2B). After 21 days of culture treated with 10 µM KGN, parallel fibres disappeared and the majority of cells acquired a cuboidal shape and displayed less-ordered extensive stress fibres (Figure 2B). Identically, hBMSCs cultured under defined chondrogenic conditions showed an extensive reorganization of actin filaments in comparison with undifferentiated cells.

### 2.3. Immunofluorescence Staining of hBMSCs after KGN Treatment in Monolayer Culture

To evaluate chondrogenic differentiation in monolayer, indirect immunofluorescence staining for a cartilage-specific markers was performed on day 21. The effect of KGN on cartilage-specific markers are shown in Figure 2C,D. Collagen II and aggrecan are the major structural components of articular cartilage. The expression of these markers was observed in culture of hBMSCs with 10 µM KGN and in culture with defined chondrogenic medium and were mainly distributed in the extracellular matrix (ECM). They were barely detectable in the control group. Immunofluorescent staining for collagen I and osteocalcin indicates the absence of hyperthrophic differentiation and endochondral bone formation (Figure 2E,F). However, hBMSCs cultured in culture medium alone showed a weak presence of collagen I in comparison with cells treated with KGN or chondrogenic medium, respectively.

### 2.4. Attachment and Proliferation of hBMSCs on OA Cartilage Surface

**Figure 3:**
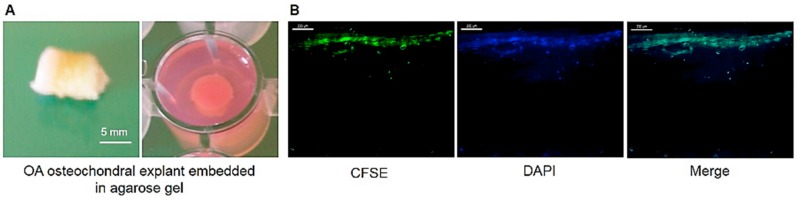
(**A**) Representative macroscopic image of the OA osteochondral explant; and (**B**) a representative fluorescence photomicrograph of cryo-sections showing attachment and proliferation of CFSE-labelled hBMSCs on the surface of OA explant on day 21 of co-culture. hBMSCs are indicated by green fluorescence and cell nuclei are stained with DAPI (blue). Scale bar represents 200 µm.

The attachment and survival of hBMSCs on the OA cartilage surface during co-culture experiments based on the use of OA osteochondral explants (Figure 3A) embedded in agarose gel was monitored by fluorescent microscopy using CFSE-labeled hBMSCs before co-culture. The layer of hBMSCs on top of the cartilage shows the fluorescent signal at the end of co-culture experiment after 21 days (Figure 3B), therefore indicates that the majority of seeded cells attached and proliferate on the OA cartilage surface in vitro.

### 2.5. Scanning Electron Microscopy (SEM) Analysis of hBMSCs’ Morphology upon Adhesion and Chondrogenic Differentiation on OA Osteochondral Explants

**Figure 4:**
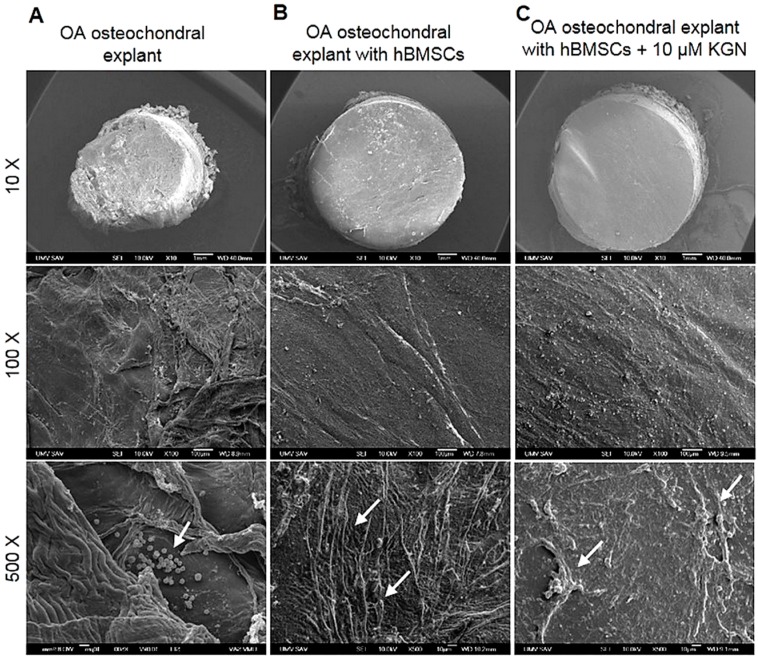
Scanning electron microscopy (SEM) at different magnifications showing the surface of (**A**) OA osteochondral explants alone (white arrows indicate inflammatory cells) and after 21 days of co-cultivation with (**B**) hBMSCs (white arrows indicate the typical spindle-shape morphology of cells) and with (**C**) hBMSCs with 10 µM KGN (white arrows indicate spherical aggregates).

OA osteochondral explants with 8.0 mm thickness and 6 mm in diameter, alone or in co-culture with hBMSCs, with/without 10 µM KGN, were examined by SEM during 21 days of culture. As revealed by SEM, the OA cartilage surface without cells was uneven and shows a distribution of different diameters of collagen fibres (Figure 4A). Fibre breakdown induced remodelling of the collagen meshwork. As shown in Figure 4A, free cellular elements and blood components, including mainly lymphocytes, monocytes, and macrophage-like cells, are presented on the damaged OA cartilage surface, while explants co-cultivated with cells with/without KGN exhibited a smoother surface without the presence of distinguishable immune cells (Figure 4B,C). After 21 days of co-culture, SEM observation revealed that the cells have firmly attached to the cartilage surface, forming interconnections and monolayers. hBMSCs without KGN treatment showed typical morphology with elongated spindle shapes (Figure 4B). hBMSCs cultured with KGN also showed a higher number of cells at the end of culture period (Figure 4C), but with forming more clusters, indicating that hBMSCs could be in an early state of chondrogenic differentiation.

### 2.6. Histologic Evaluation of OA Osteochondral Explants during Chondrogenesis in a Co-Culture 3D System

**Figure 5:**
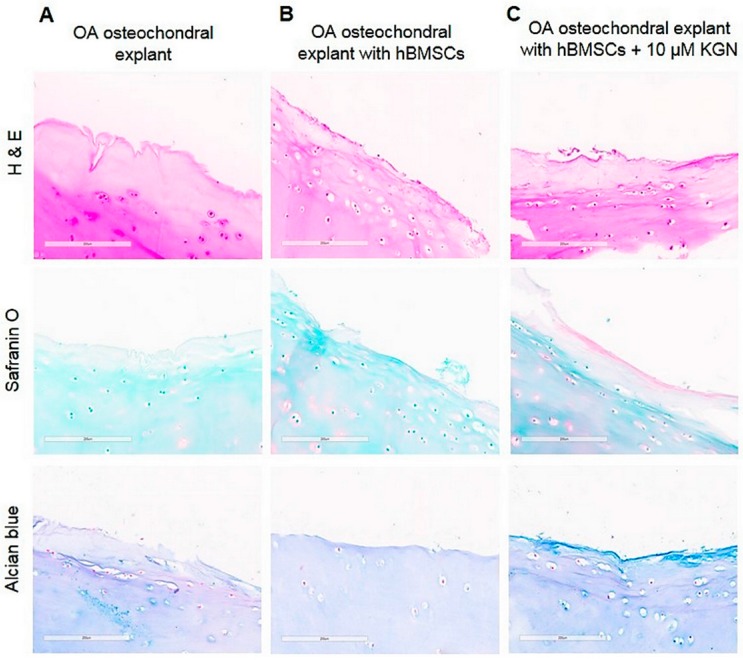
Histologic evaluation of OA cartilage surfaces of (**A**) the explant alone, without cell seeding; (**B**) the hBMSC seeded explant; and (**C**) the hBMSC seeded explant supplemented with 10 µM KGN after in vitro cultivation for 21 days. The scale bar represents 200 µm.

To evaluate the behaviour of hBMSCs in co-culture experiments on OA cartilage surface with/without KGN, the osteochondral explants were analysed by histology. As shown in Figure 5, the matrix integrity of the cartilage seemed to be largely unaffected during the whole culture period in all groups. After three weeks of co-culture the thin layer of colonized hBMSCs was observed on the cartilage surface, suggesting the ability of cells to adhere and proliferate with/without KGN (Figure 5B,C). The weak degree of Safranin O and Alcian blue staining was observed in OA cartilage without cells, exclusively at the cartilage surface, indicating intensive loss of proteoglycans and glycosaminoglycans (Figure 5A). The enhanced staining for Alcian blue and Safranin-O was observed on the edge of cartilage in group treated with KGN when compared to hBMSCs in the absence of KGN (Figure 5C).

### 2.7. Quantification of Proteins in Culture Supernatants

**Figure 6:**
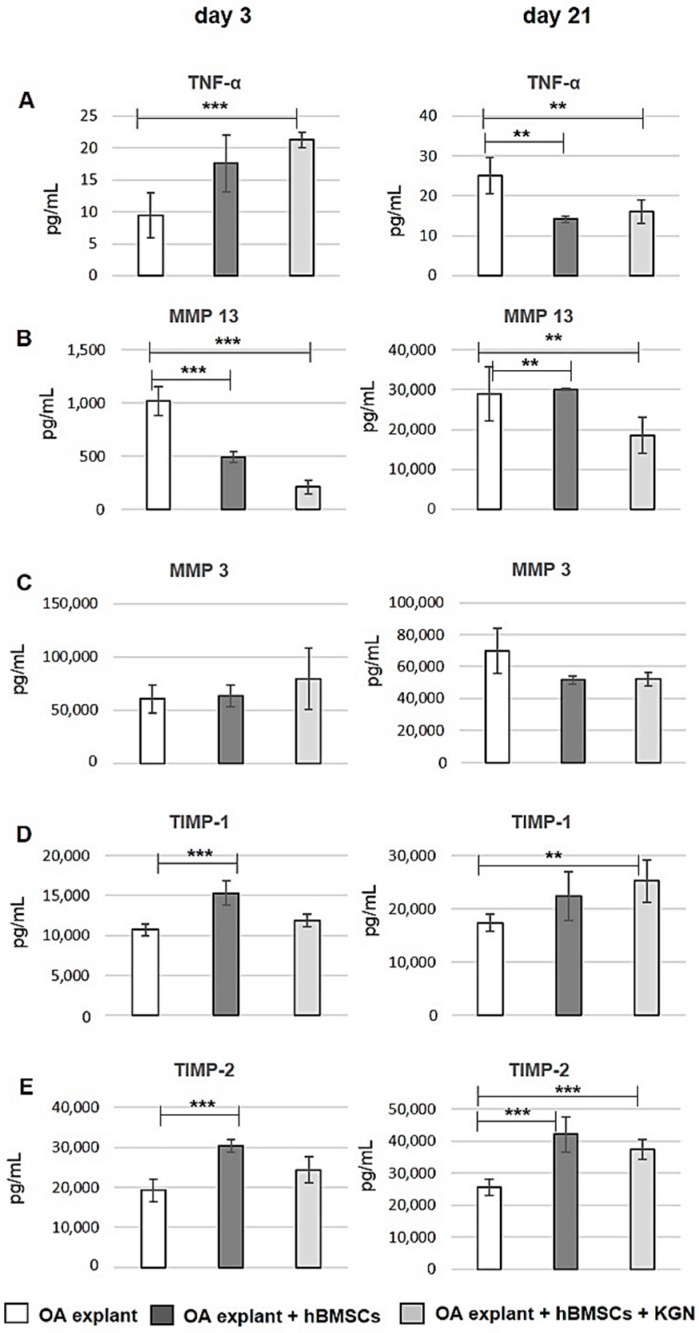
Quantification of proteins in culture supernatants. Analysis of supernatants at days three and 21 of OA osteochondral explant alone (white bars), explant seeded with hBMSCs with/without KGN (light grey/dark grey bars). Total amount of proteins in pg/mL: (**A**) TNF-α; (**B**) MMP 13; (**C**) MMP 3; (**D**) TIMP-1; and (**E**) TIMP-2 were quantified by multiplex ELISA assay (*n* = 3). ** *p* < 0.01, *** *p* < 0.001 vs. the control group (OA osteochondral explant without cell loading).

Multiplex ELISA analysis was used to quantify the level of extracellular proteins, like tissue inhibitors of metalloproteinases (TIMP), matrix metalloproteinases (MMP), and interleukins (IL) using the following panel of specific markers—TIMP-1, TIMP-2, MMP 3, MMP 13, TNF-α, IL-1β, and IL-10—produced by the cells in the co-culture experiments. As shown in Figure 6A, the content of pro-inflammatory cytokine TNF-α in the culture medium of both hBMSC groups decreased significantly (*p* < 0.01) on day 21 of the in vitro co-culture experiment after initially increasing on day 3. Meanwhile, hypertrophic marker, matrix metalloproteinase 13 (MMP 13) in the culture medium decreased significantly (*p* < 0.001 and *p* < 0.01, respectively) over time in cell-treated groups in comparison to the control without cells (Figure 6B). The secretion of another degrading enzyme, MMP 3, was not significantly affected by hBMSCs with/without KGN during the co-culture period (Figure 6C). The level of two tissue inhibitors of metalloproteinases (TIMP-1, TIMP-2) was significantly higher after hBMSCs and KGN treatment after 21 days of culture compared to control (*p* < 0.01 and *p* < 0.001, respectively), demonstrating a major contribution of the co-cultured hBMSCs to the secretion of TIMPs also in the presence of KGN (Figure 6D,E). We also quantified IL-10 and IL-1β at days three and 21 in the culture medium, but their concentration was close to the ELISA detection limit (data not shown).

## 3. Discussion

Degenerative articular cartilage, and its successful treatment, remain an insurmountable problem and challenging issue for clinicians worldwide. Among a number of therapeutic strategies, the use of chondrogenically-differentiated MSCs has been proposed as a therapeutic strategy for promoting cartilage regeneration. Here we investigated the influence of KGN on chondrogenic differentiation of human BMSCs in monolayer culture and on OA osteochondral explant in vitro. Initial attempts were made to culture hBMSCs with cartilage as a complete construct to create more closely the in vivo environment, because it is important to understand the effect of the OA environment and cell-to-cell contact on the behaviour of hMSCs. The agarose embedded OA osteochondral explant model was performed to ensure the reliable fixation of the explant on the bottom of holes and to create a defined space above the cartilage for precise seeding of hBMSCs on the cartilage surface, similar to the study of Pretzel et al. [18]. Precise location and subsequent proliferation of CFSE-labelled hBMSCs on the surface of the OA osteochondral explant was observed during 21 days of co-culture experiment, indicating the applicability of our co-culture model for further study of cell-based cartilage repair strategies in vitro. 

Our findings reported in this study show that the small drug-like molecule, KGN, stimulates cell proliferation, and induces in vitro chondrogenic differentiation of hBMSCs alone in monolayer culture and in co-culture with OA osteochondral explant. Molecules that activate the chondrogenic potential of MSCs may potentially prevent further cartilage destruction and stimulate repair of cartilage lesions. Small molecules have beneficial properties, including rapid diffusion across cell membranes, temporal regulation of protein function, specifically targeting the signalling pathway, and they are easier to obtain and can be well controlled [19]. Kartogenin was found to have long-term activity on chondrogenesis in human and rodent modesl, possess chondroprotective activity in bovine chondrocytes, minimally induce unwanted chondrocytes proliferation, have good joint retention, and is subject to rapid systemic clearance. 

Several in vitro and in vivo studies reported that KGN can modulate chondrogenesis. In the study by Ono et al. [11], KGN prevented IL-1β-induced damage to chondrocytes, neocartilages, and cartilage explants due to chondroprotective effects with no significant changes in the anabolic markers of cartilage tissue, such as collagen II, aggrecan, and Sox 9. A study conducted on bone marrow MSCs and chondrocytes from Sprague-Dawley rats demonstrated that KGN, in combination with transforming growth factor-β1 (TGF-β1) and bone morphogenetic protein-7 (BMP-7), not only triggered chondrogenesis of MSCs, but also enhanced the production of lubricin from chondrocytes [20]. Kang et al. conjugated KGN with chitosan for an intra-articular KGN delivery system, which exhibited drug release for up to seven weeks in surgically-induced OA rat models [13]. In a mouse model of OA, intra-articular injection of KGN resulted in a decrease in superficial and midzone cartilage fibrillation, reducing of subchondral bone changes, and diminished pain. Recently published animal in vivo studies in a rabbit model of OA showed that KGN may prove a useful agent to treat full-thickness cartilage defects in rabbits by direct intra-articular injection of 10 μM KGN [15], by utilizing KGN-incorporated thermogel to support bone marrow MSCs [16] or by application of KGN incorporated into a poly(lactic-co-glycolic acid) (PLGA) scaffold and stabilized with photo-cross-linkable acrylated hyaluronic acid [21]. The most recent study on the treatment of tendon-bone junction injuries in a rat tendon graft-bone tunnel model was carried out by injection of KGN with PRP as a carrier. Promising results indicate that the combination of KGN-PRP promotes the formation of the fibrocartilage zone between the tendon graft and bone interface [22].

Human BMSCs isolated from bone marrow aspirated from the proximal metaphysis of the tibia during total knee replacement fulfilled the minimal criteria defined by the ISCT for identification of MSCs [23]. The phenotype and differentiation potential of BMSCs from tibia are similar to those of bone marrow MSCs from the iliac crest as it was shown in several other studies. The difference was described mainly in the concentration of obtained progenitor cells [24]. However, the activity of hBMSCs obtained from OA patients is controversially described in the literature [25,26]. Despite these discrepancies being related to the differentiation capacity of OA BMSCs, the choice of these cells in our co-culture experimental model was adequate. It is important to highlight that these cells are derived from OA patient who should be treated in the future with similar stem cell therapies, so our model co-culture system provided more complexity than cell culture alone with mimicking the microenvironment related to OA.

Our results showed that hBMSC proliferation was enhanced by KGN, and no cytotoxic effect on these cells in vitro were observed in the concentration range of 0.1–100 µM. Among the four different concentrations, that of 10 µM contributed to the highest rate of proliferation during nine days of culture, while 100 µM concentration resulted in reduced proliferation rate (Figure 1). The tested concentrations of KGN used in this study were based on evidence for bioavailability and bioactivity in previous studies by several groups [13].

In this study we have demonstrated, that hBMSCs in monolayer culture were intensively stained for chondrogenic differentiation markers, collagen II and aggrecan, after KGN treatment (Figure 2C,D). In contrast to most published studies [27,28], the expression of cartilage-specific genes, collagen II and aggrecan, was weakly detected in undifferentiated hBMSCs at the end of monolayer culture in this study. Collagen II represents the predominant collagen in hyaline cartilage and is considered to be the most important marker of chondrogenesis. Our surprising result related to the weak detection of collagen II in undifferentiated hBMSCs could be due to the OA nature of source from which cells were isolated. The constitutive expression of aggrecan was also presented in other studies, concluding that aggrecan may not always be a sensitive marker of chondrogenesis [29]. These results are based only on the detection of proteins on the translation level, but the presence of mRNA and gene expression was not explored in this study. The production of collagen I was weakly detected in monolayer culture in the control group without chondrogenic induction (Figure 3E). The continual expression of collagen I in chondrogenic differentiated MSCs was described in previous studies [29,30]. Furthermore, collagen I is described as a component of the fibrocartilaginous tissue that is usually generated by bone-marrow cells recruited by penetrating subchondral bone. Thus, we suppose that our result may be related to the fact, that hBMSCs used in our experiments were obtained from an OA patient. In addition to cartilage-specific markers, a bone-specific marker, osteocalcin, was analysed. The detection of specific osteogenic markers in monolayer primary hMSC culture was also reported by others, showing the presence of a significant level of osteogenic commitment in undifferentiated cells [31]. Despite of these findings, during cultivation of hBMSCs in monolayer, the expression of osteocalcin was not detected neither in undifferentiated hBMSCs nor in cultures with KGN or defined chondrogenic medium (Figure 3F). Data from several other studies suggest that human MSCs alter their cytoskeletal components during differentiation [32]. These cytoskeletal rearrangements were also observed during chondrogenic differentiation by KGN in monolayer culture (Figure 2B).

Induction and expression of several chondrogenic markers was reported in different studies by treatment of BMSCs with growth factors like TGF-β, bone morphogenetic protein (BMP), insulin-like growth factor, and also by paracrine factors released from co-cultured articular cartilage or chondrocytes [17,33]. Only a few studies have examined the influence of OA cartilage ECM and OA chondrocytes on the behaviour and chondrogenic differentiation potential of co-cultured cells. To our knowledge, this is the first study indicating that bioactive factors secreted by OA BMSCs in the presence of KGN have anti-inflammatory and anti-catabolic effects in OA osteochondral explants in vitro. In our present study, we analysed proteins involved in OA pathology and known to be produced by cartilage in the culture medium. The pro-inflammatory cytokines TNF-α and IL-1β released during OA act on chondrocytes and inhibit the production of collagen II and aggrecan, which are the major components of ECM. In addition, they increase the level of matrix metalloproteinases (MMPs) and other catabolic enzymes that degrade the matrices, resulting in cartilage loss and degeneration. The pro-inflammatory environment can modulate some of the essential characteristics of MSCs. Several studies have described the negative effect of pro-inflammatory cytokines on the MSCs chondrogenic differentiation [34]. In our experimental study, we observed that co-culture of hBMSCs with OA osteochondral explant clearly decreased the level of TNF-α in culture medium with/without KGN (Figure 6A). Additionally, to monitor a degradation process in response to seeding of hBMSCs, the concentration levels of MMPs were evaluated in culture medium. It is well known that MMPs have been implicated in participating in controlling MSC differentiation [35]. MMP 3 has been shown to be strongly expressed in normal and early degenerative cartilage and decreased in osteoarthritic cartilage. In contrast, MMP 13 revealed increased expression levels and activities in late-stage OA, contributing to catabolism and articular cartilage destruction. MMP 13 is a well-characterised enzyme and is produced in human chondrocytes and hypertrophic MSCs. Furthermore, MMP 13 is the dominant proteolytic enzyme in hypertrophic cartilage during endochondral ossification [36]. Therefore, considering the significantly decreased level of this hypertrophic marker in culture medium during 21 days of co-culture, BMSCs seeded on the OA osteochondral explant in the presence of KGN probably maintained the chondrocyte phenotype (Figure 6B). In contrast, high levels of MMP 13 were detected after three days of culture in culture medium from OA osteochondral explants without cells and increased during 21 days of culture. The secretion of MMP 3 was unaffected during co-culture in all groups and changes in concentration level were not significant among each group. TIMPs were specifically identified as factors secreted mainly by MSCs and play an important role in protection of matrix molecules from MMPs mediated degradation. TIMPs, which can regulate ECM remodelling and the activities of growth factors and their receptors, have also been shown to be expressed in human cartilage [37]. The biological effect of hBMSCs treated with KGN on OA osteochondral explants in our co-culture experiments was associated with a significantly higher production of TIMP-1 and TIMP-2 in comparison with explants without cells (Figure 6D,E).

Furthermore, BMSC morphology was analysed on OA osteochondral explants by SEM demonstrating the ability of cells to colonize the fibrillated surface and spread over the articular surface (Figure 4). Moreover, a typical morphological change from a fibroblast-like elongated shape to a multi layered aggregates occurred after chondrogenic induction of hBMSCs by KGN at day 21 of co-culture, suggesting the hBMSCs acquired chondrocyte phenotypes. When no stimulus was used in the co-culture experiment, the cells had a fibroblastic morphology, indicating that no differentiation occurred during the culture period of 21 days.

Results from the histological staining of OA cartilage seeded with hBMSCs and supplemented with KGN show several changes as compared with explants without KGN (Figure 5) supporting our findings from the SEM analysis. OA cartilage surfaces without cells showed a disappearance of cell organelles and the presence of necrotic cells, as well as a loss of PGs and GAGs. In contrast to our preliminary results demonstrating the initial production of PGs and GAGs on the OA cartilage surface after 21 days of co-culture with hBMSC and KGN there are several studies demonstrating the inhibitory effect of diseased cartilage and OA chondrocytes on chondrogenic differentiation of BMSCs in vitro [17,38]. We suggest that our preliminary results from histology need to be further confirmed by molecular evidence.

There are still some limitations for the present study. Our study is limited to three weeks. Perhaps prolonged culture period may be required to detect higher degrees of differentiation and production of ECM by hBMSCs on osteochondral explants. hBMSCs used in this study came from only one patient in order to maintain a homogeneous population of MSCs isolated from bone marrow, which were used in each experimental group. Samples from different patient donors might have different efficiency, proliferation capacity, and potential for differentiation. The chondrogenic ability of hBMSCs in OA cartilage is poorly understand. Evaluation of cellular behaviour on OA osteochondral explants, keeping its original 3D matrix environment, may positively reflect the in vivo cellular bioactivity and in vivo microenvironment. Another limitation of our study was the use of restricted methods to evaluate the fate of cells and OA cartilage in co-culture experiments. The use of other investigative tools, based mainly on the molecular level could strengthen our results. 

In summary, we showed, in both monolayer and co-culture studies, in vitro, that KGN had no toxic or inhibitory effect on human BMSC proliferation. We established an in vitro co-culture system where hBMSCs from OA patient were co-cultured with OA osteochondral explants. According to our results, we suggest that the production of PGs and GAGs in the presence of KGN was positively affected in co-cultured hBMSCs with OA osteochondral explants. Furthermore, KGN treatment had no significant effect on hypertrophic differentiation of hBMSCs in monolayer culture confirmed by negative staining for collagen I and ostecolacin, and in the co-culture study by detection of a decreased level of MMP 13.

## 4. Materials and Methods

### 4.1. Ethics Statement

This study was approved by the Institutional Ethics Board of Louis Pasteur University Hospital, Kosice, Slovakia. Samples were taken with patients’ written consent according to the guidelines of the local Ethics committee.

### 4.2. Preparation of Human Osteochondral Cylinders

Human osteochondral cylinders were collected from surgically-removed joints of patients undergoing total knee replacements due to OA. Using the Single Use OATS (Osteochondral Autograft Transfer System) a total of 18 specimens (nine from each donor) of similar OA grade were harvested. The osteochondral explants were transported to the cultivation lab in sterile transport medium containing high-glucose Dulbecco’s modified Eagle’s medium (DMEM) (Sigma Aldrich, Saint Louis, MO, USA) and 2% (*v*/*v*) antibiotic/antimycotic solution (10,000 units penicillin, 10 mg streptomycin and 25 μg amphotericin B per mL) (Sigma Aldrich, Saint Louis, MO, USA). The osteochondral explants from each donor were randomly distributed to experimental groups: (i) control group without hBMSCs and KGN; (ii) with hBMSCs without KGN; and (iii) with hBMSCs and KGN. For embedding of the explants, 2% agarose (Sigma Aldrich, Saint Louis, MO, USA) was filled into the wells of a 24-well plate. The osteochondral explants of a defined size (8.0 mm thick and 6.0 mm in diameter) and with cartilage surface upside were embedded in the performed gap created by inserting punch biopsy (6.0 mm) into the solid agarose gel (Figure 3A). Afterwards, the wells were filled with 800 μL of co-culture medium containing Dulbecco’s Modified Eagle’s Medium/Nutrient Mixture F-12 Ham 1:1 (DMEM/F12) (Sigma Aldrich, Saint Louis, MO, USA) supplemented with 5% (*v*/*v*) foetal bovine serum (FBS) (Sigma Aldrich, Saint Louis, MO, USA), 2% (*v*/*v*) Insulin-Transferrin-Selenium-A supplement (ITS-A) (Sigma Aldrich, Saint Louis, MO, USA) and 1% (*v*/*v*) antibiotic/antimycotic solution (Sigma Aldrich, Saint Louis, MO, USA) and cultured at 37 °C in a humidified 5% CO_2_ atmosphere. Culture media were changed two times a week and culture supernatant was removed, pooled, and stored at −80 °C for further analysis. 

In each experimental group, triplicates were cultured for each donor, and samples were analysed histologically, and processed for scanning electron microscopy (SEM) analysis. Secretome analysis of the supernatant was performed by multiplex enzyme-linked immunosorbent assay (ELISA).

### 4.3. Isolation, Expansion, and Characterization of Human Bone-Marrow Derived Mesenchymal Stromal Cells (hBMSCs)

Bone marrow aspirated from the proximal tibia was used to isolate hBMSCs from one donor undergoing total knee replacement due to OA. A previously-described method based on red blood cell (RBC) lysis with ammonium chloride was used to isolate and expand cells in culture [39]. After lysis, cells were collected by centrifugation at 150× *g* for 7 min at 4 °C, rinsed twice with DMEM and resuspended in complete culture medium containing Minimum Essential Medium Eagle (Alpha-MEM) (Alpha modification, without l-glutamine) (Sigma Aldrich, Saint Louis, MO, USA) supplemented with 10% (*v*/*v*) foetal bovine serum (FBS) (Sigma Aldrich, Saint Louis, MO, USA) and 1% (*v*/*v*) antibiotic/antimycotic solution, seeded at a density of 2.0 × 10^3^ cells/cm^2^ and allowed to become adherent. Nonadherent cells were removed after five days and the medium was changed every three days until the cells reached 80% confluence. Confluent cultures were dissociated with trypsin-EDTA (Sigma Aldrich, Saint Louis, MO, USA) and seeded at a density of 2.0 × 10^3^ cells/cm^2^. Cells from passage 2 were characterized and used for further experiments. Immunophenotype characterization of hBMSCs was performed by flow cytometry using the Human MSC Phenotyping Kit (MACS; Miltenyi Biotec, Auburn, CA, USA). Cells were immunofluorescently stained for CD14, CD20, CD34, CD45, CD73, CD90, and CD105. To evaluate multilineage differentiation of hBMSCs cells in vitro, the Human Mesenchymal Stem Cell Differentiation Kit (STEMPRO^®^, GIBCO, New York, NY, USA) was used prior to appropriate staining with Oil red O, Alcian blue, and Alizarin red, respectively.

### 4.4. Cell Proliferation Assay

The effect of KGN on hBMSCs proliferation was monitored using the xCELLigence Real-Time Cell Analysis (RTCA) system (Roche Applied Science, Penzberg, Germany). The procedure was carried out as previously described in our in vitro study [40]. Kartogenin (Sigma Aldrich, Saint Louis, MO, USA) used in this study was dissolved in dimethyl sulfoxide at a concentration of 5 mM. Different concentrations of KGN (0.1; 1; 10; 100 µM) were tested in order to determine the optimal concentration for our experiments. In brief, 3000 cells/well in 100 μL of culture medium (Alpha-MEM, 10% FBS, 1% ATB) in E-Plate 16 was exposed after 24 h to 100 μL of medium containing indicated concentrations of KGN. Controls received either culture medium only with 10% FBS. The impedance value of each well was monitored every hour and expressed as a cell index (CI) value. The experiments were performed in quadruplicates and impedance profiles were recorded continuously over nine days.

### 4.5. Chondrogenic Differentiation In Vitro

To establish hBMSCs chondrogenesis in monolayer culture, cells from passage 2 were seeded onto 24-well plates at a density of 5 × 10^3^ cells/well. The cells were divided into three groups and the experiment was performed in duplicates: (i) control group—cultured with culture medium only (Alpha-MEM, 5% FBS, 2% ITS-A, 1% ATB; (ii) KGN group—cultured with culture medium supplemented with 10 µM KGN; and (iii) chondrogenic group—cultured with STEMPRO Chondrogenesis Differentiation Kit (GIBCO, New York, NY, USA) in accordance with the manufacturer’s instruction. The medium was changed every three days and cells were cultured for up to three weeks. After 21 days, cells were processed for cytoskeletal and immunofluorescence staining as described below.

In order to determine the effect of KGN on chondrogenic capacity of hBMSCs, cells were co-cultured with OA osteochondral explants for 21 days in three groups mentioned above. Co-culture medium was gently removed from the OA cylinder embedded in agarose gel and cell seeding was performed by a local adherent technique in a dropwise manner. Before co-culture experiments, hBMSCs were cultured for 24 h in a medium mixture containing equal parts of culture medium (Alpha-MEM, 10% FBS, 1% ATB) and co-culture medium (DMEM/F12, 5% FBS, 2% ITS-A, 1% ATB). Suspension of hBMSCs (1 × 10^5^ cells/explant) in a volume of 50 µL of co-culture medium was carefully added onto the cartilage surface. The additional volume of co-culture medium (750 µL) was added after initial attachment of cells. Co-culture medium was supplemented with 10 µM KGN in one group. Cell-free OA cylinders were used as controls. Every three days, 800 μL of the culture supernatants were carefully removed for analysis and replaced with fresh co-culture medium with/without KGN. 

In each experimental group, three cylinders from each donor (*n* = 2) were cultured. A total of three samples from each experimental group were processed for SEM analysis after 21 days of co-culture, and three samples were analysed histologically. Supernatants on day 3 and 21 were used for multiplex ELISA.

### 4.6. Cytoskeleton and Adhesion of hBMSCs

To observe the cytoskeleton and cell morphology after KGN treatment in monolayer culture, hBMSCs were stained with TRITC-conjugated Phalloidin (Millipore, Burlington, MA, USA) according to manufacturer’s instruction. Briefly, cells were fixed with 4% paraformaldehyde in PBS for 15 min and permeabilized with 0.5% (*v*/*v*) Triton-X 100 (Sigma, Cream Ridge, NJ, USA) in PBS for 5 min prior to staining. After, 1% (*v*/*v*) of bovine serum albumin (BSA) (Sigma Aldrich, Saint Louis, MO, USA) was used to block the non-specific binding. Finally, cells were stained with optimal concentration of TRITC-conjugated Phalloidin for 30 min.

To confirm the attachment and proliferation of hBMSCs on the cartilage surface of OA osteochondral explant, the cells were labelled before seeding with 5(6)-carboxyfluorescein diacetate *N*-succinimidyl ester (CFSE) (Sigma Aldrich, Saint Louis, MO, USA) according to the supplier’s instructions. hBMSCs from passage 2 were trypsinized, labelled, and seeded at a density of 1 × 10^5^ cells/explant in 50 µL co-culture medium onto the surface of explant prepared as described above and allowed to attach for 10 min. The additional of 750 µL fresh culture medium was added after initial cell attachment and seeded explant was incubated in a humidified atmosphere at 37 °C with 5% CO^2^. For the detection of labeled hBMSCs after 21 days of co-culture, OA explant was fixed with 4% buffered paraformaldehyde overnight at 4 °C, rinsed in 30% sucrose and embedded in OCT-freezing medium. Cryostat sections (50 µm) were prepared and the cell nuclei were stained with 4,6-diamidino-2-phenylindole (DAPI) for 10 min. Tissue sections were immediately assessed using a fluorescent microscope (Leica, DMi3000B, Wetzlar, Germany).

### 4.7. Immunofluorescence Staining

The production of collagen II, aggrecan, collagen I and osteocalcin by hBMSCs in monolayer culture was visualized through immunofluorescence after three weeks of chondrogenic culture. Cells on 24-well plates were fixed with 4% formaldehyde in PBS for 15 min after achieving 60–80% confluency and permeabilized by 0.5% Triton X-100 for 30 min at 37 °C followed by 10 min at room temperature. After blocking with 10% normal goat serum in 0.1% Triton X-100 diluted in PBS for 1 h, cells were labeled overnight at 4 °C with an antibody against Collagen II, Aggrecan, Collagen I and Osteocalcin. For fluorescence detection, samples were incubated for 1 h with the respective secondary antibodies. Antibodies were purchased from Abcam (Cambridge, UK). Finally, the nuclei was stained with DAPI and samples were analysed with an inverted fluorescence microscope (Leica, DMi3000B, Wetzlar, Germany).

### 4.8. Histological Assay

In order to evaluate the adherence and chondrogenic differentiation potential of hBMSCs on OA osteochondral explants upon stimulation with KGN, histological staining was performed. Prior to staining, cartilage sections after removal of bone were fixed in formalin, decalcified, embedded in paraffin using standard protocols, and then 4-μm thick sections were cut from the central part of the cartilage disc. After deparaffinization in xylene for 30 min, sections were rehydrated and cartilage morphology was analysed by hematoxylin/eosin staining. Glycosaminoglycan (GAG) content was assessed by Alcian blue staining combined with counterstaining of iron hematoxylin to observe nuclei and proteoglycans (PGs) were stained with Safranin O and counterstained with Fast green. All images were obtained using a high-throughput Aperio AT2 Digital Pathology Scanner automated digital image system (Leica, Wetzlar, Germany).

### 4.9. Scanning Electron Microscopy Analysis

The characteristics of the OA cartilage surface and cell morphology with/without KGN on day 21 of co-culture experiments were observed under scanning electron microscopy (SEM). In order to prepare samples for SEM, the specimens were fixed in a mixture of 25% glutaraldehyde, 10% paraformaldehyde and 0.2 M sodium cacodylate buffer (pH 7.2) (Merck-Darmstadt, Hohenbrunn, Germany) for 48 h at 4 °C. The specimens were subjected to sequential dehydration with a gradient ethanol, followed by two washes with hexamethyldisilazane for 15 min. After drying, the specimens were vacuum-coated with gold and then observed by a scanning electron microscope JEOL JSM-7000 F (JEOL Ltd., Akishima-shi, Japan).

### 4.10. Analysis of Culture Supernatants by Multiplex ELISA Assay

Culture supernatants were analysed in order to determine the level of secreted proteins during chondrogenic diffrentiation of hBMSCs with/without KGN on OA cartilage surface. The concentration of MMP 3, MMP 13, TIMP-1, TIMP-2, TNF-α, IL-1β, and IL-10 was measured by using the multiplex ELISA Quantibody Human Cytokine Antibody Array (RayBiotech, Baria, Prague, Czech Republic). Assays were performed according to the manufacturer’s instructions, and data were obtained from experiments in triplicate. 

### 4.11. Statistical Analysis

All values were expressed as the mean ± standard deviation of the mean (SD). Statistical significance was determined by *t*-test and results were considered to be significant if *p* < 0.01 (**) and *p* < 0.001 (***).
